# Molecular Identification of *Bacillus* Isolated from Korean Water Deer (*Hydropotes inermis argyropus*) and Striped Field Mouse (*Apodemus agrarius*) Feces by Using an SNP-Based 16S Ribosomal Marker

**DOI:** 10.3390/ani12080979

**Published:** 2022-04-10

**Authors:** Md-Mafizur Rahman, Sang-Jin Lim, Yung-Chul Park

**Affiliations:** 1 Division of Forest Science, Kangwon National University, Chuncheon 24341, Korea; mmrahman@btge.iu.ac.bd; 2 Department Biotechnology and Genetic Engineering, Faculty of Biological Science, Islamic University, Kushtia 7003, Bangladesh; 3 Institute of Forest Science, Kangwon National University, Chuncheon 24341, Korea

**Keywords:** *Bacillus cereus*, allele, 16S ribosomal RNA, single-nucleotide polymorphism

## Abstract

**Simple Summary:**

Wildlife is a great concern because of its free-ranging movements. They carry bacterial zoonoses in their feces, such as *Bacillus* species. In this study, we developed a 16S *Bacillus* species-specific 16S ribosomal RNA (rRNA) molecular marker for species identification. For discrimination of genetically similar members of *Bacillus* *cereus* group, including *Bacillus cereus, B. anthrax*, and *B. thuringiensis*, a single nucleotide polymorphism (SNP)-based marker was developed. We altered an artificial base at the 3′-end of SNP sites in each SNP-based 16S rRNA primer sequence to improve the ability of SNP-based primers to bind the PCR template sequence, thereby improving the allele-specific detection of target *B. cereus* strains. SNP analysis in high-quality DNA sequences could facilitate identification and discrimination of closely related bacterial species.

**Abstract:**

Ambiguous, heterogeneous, endospore-forming *Bacillus* species, notably *Bacillus cereus*, often produce fatal toxins that threaten human health. We identified *Bacillus* from wild animal fecal samples (*n* = 80), including the Korean water deer (*n* = 25) and striped field mouse (*n* = 55). Using traditional culture-based methods, 25 animal fecal samples (31.25%; 25/80) were found to be positive for *Bacillus* species, whereas using molecular techniques, 19 samples (23.75%; 19/80) were found to be positive for the same. In addition, we designed a *Bacillus* species-specific 16S ribosomal RNA (rRNA) gene marker and utilized it to identify 19 samples by means of PCR amplification and sequencing, using at least one colony from the 19 *Bacillus* positive samples. The recovered sequences were matched to sequences of three *Bacillus* species (*B. cereus*, *B. amyloliquefaciens,* and *B. megaterium*) from the GenBank database. Moreover, the phylogenetic tree generated in this study established specific clades for the *Bacillus* group. In addition, to differentiate between *B. cereus*, *B. anthracis*, and *B. thuringiensis*, we designed a single nucleotide polymorphism (SNP)-based primer by identifying SNPs in the alignment of 16S rRNA gene sequences of *B. cereus* group strains. The SNPs were used to design primer sets for discrimination between highly similar species from the *B. cereus* group. The study could be used in surveillance of agricultural fresh-produce-associated *Bacillus* outbreaks, for accurate identification of each *Bacillus* species, and in the development of control measures.

## 1. Introduction

Water deer (*Hydropotes inermis argyropus,* the most common ungulates) and striped field mice (*Apodemus agrarius*) are widely found in Korea [1]. Based on a National Institute of Biological Resources (NIBR) report [2], the population of Korean water deer is increasing, which has led to a food shortage and an increase in the likelihood of them coming into contact with people and livestock [3]. Several reports have suggested that zoonotic pathogens, including viruses [4,5], bacteria [3,6], pests [7], and mites [8], can be transmitted to humans and animals from wild animals, including *H. i. argyropus* and *A. agrarius*. Research [9] has shown that a higher percentage of bacterial zoonotic diseases than viral diseases occurs in wild animals. In addition, the prevalence of pathogenic *Bacillus* spp., including *B. cereus*, *B. anthracis,* and *B. thuringiensis*, is high in animal feces [10,11,12].

*Bacillus*, which consists of large, heterogeneous, aerobic/anaerobic, motile, spore-forming, and rod-shaped bacteria, is widely distributed in nature, including in animal feces. They may cause fatal diseases in humans by producing emetic toxins, hemolysins, and other potential virulence factors [13,14]. To date, over 2446 *Bacillus* species and 247 *Bacillus* genera (https://lpsn.dsmz.de/genus/bacillus.html, accessed on 24 January 2022) have been retrieved from different environmental sources as well as wild and domestic animals, insects, and animal feces [15,16,17]. Due to their different ecological lifestyles, *B. cereus* group species generally live in diverse environments, such as mammalian intestines, soils, and plants [18]. The pathogenic *Bacillus* species spread from feces to different environmental sources and survive for a long period, even in harsh environments in nature [19]. Moreover, bacteria in the *B. cereus* group have a significant impact on human health. Members of the *B. cereus* group, including *B. cereus*, are the most dominant species that cause diseases in humans and animals [20]. Food poisoning caused by *B. cereus* generally results in emetic and diarrheal syndromes [21]. It causes food-borne diseases and poses a serious threat to public health. The annual prevalence of diarrheal diseases is significantly increasing worldwide [22,23]. According to a report, more than 123 *B. cereus*-caused food poisoning outbreaks have emerged in Korea from 2001 to 2019 [23], and a few of them have recorded in Korea [22,24]. Over the past 20 years, the 16S ribosomal RNA (rRNA) gene has been used as a standard for the identification of bacteria, including *Bacillus* species [25,26,27]. The 16S rRNA gene sequence is also widely used for identifying the phylogenetic relationships between known and unknown bacteria [28]. In a study, 69 *Bacillus* species approved by the International Journal of Systematic Bacteriology were identified using their 16S rRNA gene sequences. The study suggested that the 5′-end region of the 16S RNA contains a hypervariant region (275 bp) that is highly specific for *Bacillus* species. Furthermore, sequence analysis of the hypervariant region of *Bacillus* strains revealed that this region is highly conserved within the species [26] and thus can be used as an ideal index for the identification of *Bacillus* species. However, in a phylogenetic analysis, Wang et al. showed that *Bacillus* is not a monophyletic group of bacteria [29]. In fact, *B. cereus* is a highly versatile group of bacteria comprised of seven closely related species, including *B. thuringiensis, B. cereus*, *B. anthracis*, *B. mycoides*, *B. weihenstephanensis*, *B. cytotoxicus*, and *B. pseudomycoides* [14,18,30].

Single-nucleotide polymorphisms (SNPs) are important molecular markers that are increasingly being used for species- or strain-specific identification of bacterial pathogens, including *B. cereus* [31] and *B. anthracis* [32]. Due to genetic stability and low mutation rates (10^−10^ per site per generation of *Bacillus* sp. [33]), SNPs can be used for the identification of similar strains of a bacterial group, such as the *B. cereus* group. Nevertheless, studies have also found that SNPs can be used to efficiently discriminate between similar members within a specific genetic group [31]. Moorhead et al. [34] identified a few diagnostic SNPs in the RNA polymerase sigma factor (*sigB*) gene [34]. Moreover, an allele-specific PCR method with SNP markers has been used to discriminate between serotype- or lineage-specific Shiga toxin-producing *Escherichia coli* bacteria [35]. The present study introduced allele-specific alter bases at the 3′-end (SNP site) of the 16S rRNA primer sequences developed to detect *B. cereus*.

In our study, we observed high levels of sequence similarity (>99%) in the alignment of 16S rRNA gene sequences of *B. cereus, B. anthracis*, and *B. thuringiensis,* similar to the observations reported in some studies [31,36]. Identification and differentiation of the three species (*B. anthracis*, *B. cereus*, and *B. thuringiensis*) using 16S rRNA gene sequences is considered a bottleneck. Therefore, in this study, we designed an SNP-based marker from the aligned gene sequences of three *B.* cereus species that could effectively and accurately differentiate between them. Moreover, we introduced an artificial mismatch nucleotide (transversion) within the three bases closest to the 3′-end (SNP site) in the 16S rRNA primer sequence to allow for differentiation between members of the *B. cereus* group. The study aims to identify the *Bacillus* species-specific primers and SNP-based *B. cereus*-specific primers for early detection and to minimize the safety risks associated with wild-animal fecal pathogens.

## 2. Materials and Methods

### 2.1. Bacillus Species-Specific and Bacillus cereus-Specific Identification from Wild-Animal Feces

**Figure 1:**
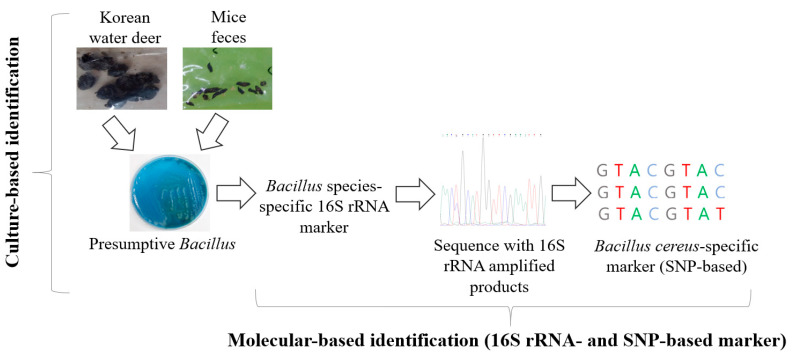
Flowchart of the overall steps involved in this study of cultural and molecular identification of *Bacillus* from wild-animal feces (Korean water deer (*Hydropotes inermis argyropus*) and striped field mouse (*Apodemus agrarius*)).

In Figure 1, we depict the overall identification process of *Bacillus* with the culture- and molecular-based approaches. The newly developed, *Bacillus* species-specific 16S ribosomal RNA gene (16 rRNA) was sequenced with amplified PCR products and matched to the GenBank database in order to identify *B. cereus* with a *Bacillus cereus*-specific SNP-based marker.

### 2.2. Sample Collection and Processing

A total of 80 animals were collected, including striped field mice (*n* = 55; *Apodemus agrarius*) and Korean water deer (*n* = 25; *Hydropotes inermis argyropus*). Fecal samples were collected from mice captured at Seoraksan National Park (38°08′52.70″~38°14′48.30″ N; 128°27′06.40″~128°33’51.12″ E), Odaesan National Park (37°24′12.21″~37°59′55.63″ N; 128°01′14.3″~128°56′41.40″ E), and Sobaekson National Park (36°57′12.60″~36°57’22.80″ N; 128°26’14.5″~128°28′11.9″ E) in Sokcho, Pyeongchang, and Yeongju, South Korea, respectively. Sample identification number, collection localities, and Global Positioning System (GPS) coordination of collection places are provided in Supplementary Table S1. In addition, each of the captured wild mice was kept in a Sherman Trap (H.B. Sherman Traps Inc., Tallahassee, FL, USA) and then released after the collection of fresh feces. Fresh feces of Korean water deer (*H. i. argyropus*) were also collected from nearby agricultural areas of the forest. The fecal color and morphology were different in each animal, and fecal samples were collected from the tested wild animals. Wild-animal feces was identified based on color and shape (https://www.discoverwildlife.com/how-to/identify-wildlife/how-to-identify-animal-droppings/, accessed on 26 March 2022). The fecal samples were brought into the laboratory in an icebox, where they were processed within three hours. Sample collections were conducted with permission and according to the guidelines of the local government. In addition, the protocol of this experiment was approved (approval number: KW210701-1) by the Animal Handling Committee according to the guidelines of the Institutional Animal Care and Use Committee of Kangwon National University, Chuncheon, South Korea.

### 2.3. Traditional Identification of Bacillus Species Using the Culture-Based Method

The fecal samples (0.1 to 1 g for mouse; 10 g for water deer) were suspended in a ratio of one-quarter-strength ringer solution (Oxoid, Basingstoke, Hampshire, UK) and mixed vigorously to make a uniform suspension. The fecal samples were then serially diluted up to 10^8^-fold, following which 0.1 mL of these aliquots were spread on triplicate agar plates of nutrient agar (NA) and tryptic soy agar (TSA), and the plates were incubated at 37 °C for 24 h, as in our previous studies [12,37]. We identified *Bacillus* spp. based on the appearance of colonies with an irregular shape, flat, gray/white, rhizoid/hairy structure, and a 2–5 mm diameter on culture media [38]. The isolated presumptive *Bacillus* colonies were then checked on *Bacillus cereus* selective agar (BCSA) and Mannitol egg yolk polymixin agar (MYP) media (Figure S1). We observed distinctive turquoise to peacock blue color with egg-yolk precipitate on the specific BCSA media and presumed it to be *B. cereus* [23]. The colonies of *B. cereus* produced pink or pink-orange color with whitish precipitate on MYP media, while the colonies of *B. amyloliquefaciens,* especially *B. subtilis,* produced yellow color on MYP agar media [38,39].

### 2.4. Genomic DNA (gDNA) Extraction

For gDNA isolation, we selected the presumed colonies grown from the feces of individual animals. A single, pure *Bacillus* isolated from animal feces was transferred to 5 mL Luria Bertani broth and incubated at 35 °C for 18 h. The fresh broth culture was then preserved for further studies in 15% glycerin stock at −80 °C.

The gDNA of *Bacillus* colonies was extracted from broth culture. For this, 1 mL of fresh broth of each *Bacillus* was centrifuged for 10 min at 7500 rpm, resulting in the formation of a bacterial pellet at the bottom of the microcentrifuge tube. The supernatant was carefully discarded, and the resuspended pellet was then lysed in 200 µL enzymatic lysis solution with 20 mg/mL lysozyme at 60 °C for 30 min. This was followed by extraction of gDNA using the Biomedic ^®^ Genomic DNA Extraction Kit (Biomedic Co. Ltd., Bucheon, Korea) according to the manufacturer’s instructions. The isolated gDNA was subjected to analysis (qualitative/quantitative) using a spectrophotometer (DS-11; DeNovix, Wilmington, DE, USA); the concentration of the extracted gDNA ranged from 14.51 to 66.55 ng/µL. It was stored at 4 °C in a refrigerator for further use in the next steps.

### 2.5. Development of a 16S rRNA Primer Set for Bacillus Species Identification

For development of a new primer for *Bacillus* species identification, 585 sequences of the 16S rRNA gene of *Bacillus* species and closely related bacterial sequences were downloaded from GenBank (https://www.ncbi.nlm.nih.gov/genbank/, accessed on 30 March 2022). The sequences were aligned and analyzed using the bioinformatics analysis programs Clustal X 2.1 [39] and BioEdit 7.2.1 [40]. The primer sets were then designed based on the aligned ribosomal gene sequences, while an ambiguous code “R’ was introduced into the forward primer, Ba_F: 5′-CGRACGGGTGAGTAACACG-3′.

**Figure 2:**
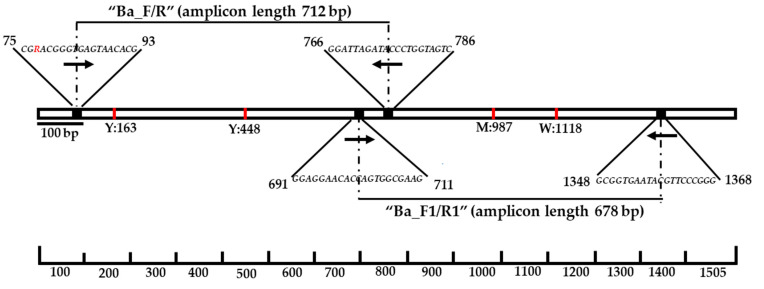
Schematic of the 16S ribosomal RNA gene (16 rRNA) sequence and Bacillus species-specific primer targets. The first primer set consists of forward primer, Ba_F (19 bp), which targeted between 75 and 93 and a reverse primer, Ba_R (21 bp), which targeted between 766 and 786 bp; the target amplicon length was approximately 712 bp. Similarly, the second primer set was Ba_F1/Ba_R1, which generated a target amplicon that was 678 bp long. The red-colored “R” indicates the bases A/G in the forward primer (Ba_F) sequence. The four natural SNP positions have been marked using a red-colored line at the positions of 163 (Y = C/T), 448 (Y = T/C), 987 (M = A/C), and 1118 (W = A/T) of the reference Bacillus (AJ000648) 16S rRNA gene sequence. The final combined (deletion of overlapping sequence) amplicon length was approximately 1293 bp.

We designed two primer pairs (Ba_F/R, amplicon length: 712 bp) and (Ba_F1/R1, amplicon length: 678 bp). Upon amplification of the gDNA of *Bacillus* using the two primers, the amplified products were sequenced and combined. Each PCR reaction consisted of 1 μL (10 ng/μL) of template DNA, 0.5 μL (10 pmol/μL) each of the forward and reverse primers, 2 μL of 10× buffer, 2 μL of dNTPs, 0.5 μL (5 unit/μL) of *Taq* DNA polymerase (Qiagen), and 13.5 μL of distilled water, in a reaction with a final volume of 20 μL. The PCR cycle conditions consisted of amplification at 95 °C for 5 min; followed by 35 cycles of denaturation for 30 s; annealing at 58 °C (for both primer sets, Ba_F/R and Ba_F1/R1) for 30 s; polymerization at 72 °C for 1 min 30 s; and a final elongation at 72 °C for 5 min. The final combined amplicon length was approximately 1293 bp, which was then sequenced. For accurate design of primers, Primer3 software v0.4.0 (http://bioinfo.ut.ee/primer3-0.4.0/, accessed on 30 March 2022) was used. The schematic diagram (Figure 2) and primer information are provided in Table 1.

### 2.6. Development of Bacillus cereus-Specific SNP-Based 16S Primer Sets

We designed SNP-based *B. cereus*-specific 16S rRNA primers for discrimination between the three members of *B. cereus* (*B. cereus*, *B. anthracis,* and *B. thuringiensis*). The 16S rRNA gene sequences of *B. cereus* strain American Type Culture Collection (ATCC) ATCC 14579 (NR074540), *B. anthracis* strain ATCC 14578 (NR041248), and *B. thuringiensis* 16S rRNA (AM779002.1) were acquired from the NCBI database (https://www.ncbi.nlm.nih.gov/ accessed on 30 March 2022). In addition, the 16S rRNA gene sequence of the isolated *B. cereus* strain (MF139612) from the *A. agrarius* fecal sample was included to design the primer set.

Four SNP positions were detected upon alignment of three NCBI-acquired (*B. cereus*, *B. anthracis*, and *B. thuringiensis*) and one laboratory-isolated *B. cereus* (MF139612) sequences, which were then used to distinguish among the members of the *B. cereus* group (Figure S2). Based on previous research, mutating the first and third position bases within the 3′-end last triplet bases of primer sets allows for very strong hybridization during PCR amplification [41,42]. Therefore, we introduced an altered base at the third position of a triplet base at the 3′-end of each primer for efficient allelic discrimination among the three strains of the *B. cereus* group (Table 1). One SNP-based primer set consisted of the forward primer Bc_F1m: 5′-GGGAAGAACAAGTGCTAGTTGYAT-3′ and reverse BCR1m: 5′-GAAGCCCTATCTCTAGGGRTT-3′, while the other primer set consisted of the forward primer Bc_F2m: 5′-CCAGGTCTTGACATCCTCTYAA-3′ and reverse primer BCR2m: 5′-GTCACCTTAGAGTGCCCAARTT-3′ (Table 1). In this study, the newly designed SNP-based 16S rRNA primer sets were used for PCR amplification performed in a reaction with a final volume of 20 μL, containing 2 μL of buffer (10×), 0.5 μL of each primer (10 pmol), 2 μL of dNTPs (2.5 mM), 0.5 μL of *Taq* polymerase (5 unit/μL), and 13.5 µL of distilled water (Dongsheng Biotech). The PCR reaction conditions were as follows: an initial denaturation for 5 min at 95 °C; followed by 35 cycles of denaturation for 30 s at 95 °C; primer annealing for 30 s at 62 °C for the primer set Bc_F1m/BCR1m and 65 °C for the primer set Bc_F2m/BCR2m; extension for 30 s at 72 °C; and a final extension for 5 min at 72 °C.

### 2.7. PCR Amplification, Followed by Sequencing and Phylogenetic Analysis

The gDNA was isolated from the Luria Bertani-cultured presumed *Bacillus*-positive colonies from animal fecal samples using the DNeasy Blood and Tissue Kit (Qiagen, Valencia, CA, USA) according to the manufacturer’s protocol. The amplified PCR products were electrophoresed on a 1.0% agarose gel and purified using a DNA Gel Extraction Kit (Qiagen). The PCR-amplified and purified 16S ribosomal gene products were sent to Biomedic Co. Ltd., Seoul, South Korea for sequencing. We checked the specificity of the designed 16S primer using five non-*Bacillus* bacterial species (*Salmonella enterica*, NCCP-15756; *Escherichia coli*, NCCP-14034; *Shigella dysenteriae,* NCCP-14746; *Enterobacter cloacae*, NCCP-10173; and *Pseudomonas aeruginosa*, NCCP-16099). Moreover, in this study, NCBI Primer-BLAST (https://www.ncbi.nlm.nih.gov/tools/primer-blast/, accessed on 30 March 2022), was used for checking the specificity of our designed primers. The multiple 16S rRNA gene sequences, especially the electropherogram of each gene sequence, were visually checked using Chromas software (Griffith University, Queensland, Australia) to identify positions with more than one peak.

The obtained bacterial 16S rRNA gene sequences were compared with other homologous sequences deposited in GenBank using BLASTN2.2.31. Phylogenetic relationships were inferred using neighbor-joining (NJ) analyses implemented in MEGA 7.0.14. The confidence of branches in ML trees was assessed using bootstrapping searches with 1000 replicates. An NJ tree was inferred using Kimura’s 2-parameter model with bootstrapping searches of 1000 replicates.

## 3. Results

### 3.1. Identification of Bacillus spp. from Wild Animal (Korean Water Deer and Striped Field Mouse) Fecal Samples

A total of 80 fecal samples from two animal species (*A. agrarius* and *H. i. argyropus*) were tested using traditional culture-based and molecular methods. The results showed the presence of *Bacillus* spp. in 19 out of the 80 animal fecal samples (Table 2). Using traditional, culture-based methods, *Bacillus* colonies were observed in 25 samples, including nine from Korean water deer and 16 from striped field mice. Based on the color of the colonies in the culture media, 25 animal fecal samples (31.25%; 25/80) were positive for *Bacillus*, while on the basis of molecular techniques such as PCR and sequencing similarity analysis, 19 samples (23.75%; 19/80) were positive.

In this study, the *Bacillus* colonies (BA#1, MF139612) showed a distinctive turquoise to peacock blue color on BCSA media with an observation of egg-yolk-like precipitation under the microscope (Figure S1A,B). The colonies produced pink or pink-orange color on the *Bacillus*-specific MYP medium with the observation of whitish precipitation as well under a high-resolution microscope (Figure S1C,D). The colonies of the *B. amyloliquefaciens* group, especially *B. subtilis*, produced a yellow color on MYP agar media (Figure S1E,F). These colonies were then amplified using the newly designed 16S rRNA primers (Table 1), following which the amplified products were sequenced.

### 3.2. Amplification of the Colonies Positive in the Culture Using The Newly Designed 16S rRNA Primers for Identification of Bacillus Species Isolated from Wild-Animal Fecal Samples

In this study, we designed two ribosomal 16S rRNA primer sets (Ba_F/R and Ba_F1/R1) based on the aligned gene sequences from GenBank (Table 1). A schematic representation of the reference *Bacillus* (AJ000648) 16S rRNA gene sequence and the positions of the forward and reverse primers are shown in Figure 2. The 25 samples found to be positive in the cultures were then amplified using these two newly designed 16S rRNA primer sets. Following that, the products amplified using the *Bacillus* species-specific primer sets were sequenced and matched to the NCBI database. Only 19 of the 25 samples were found to be PCR-amplified with the desired bands (Table 2).

**Figure 3:**
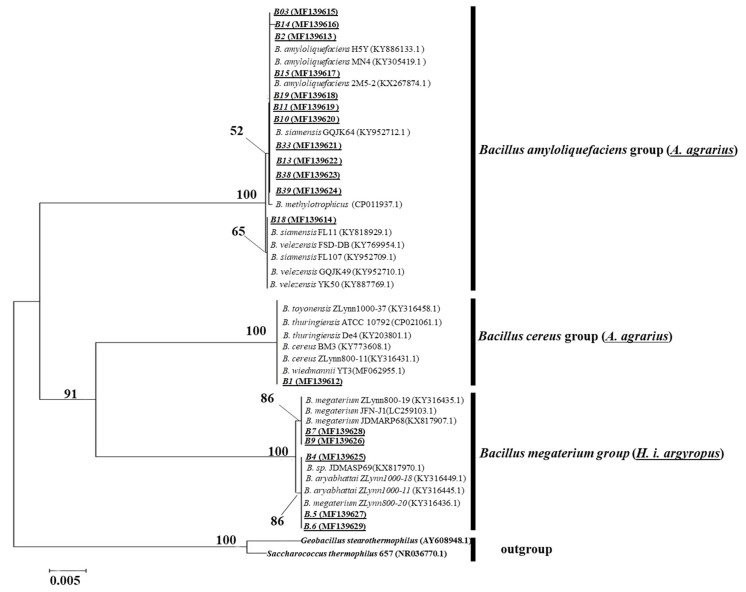
Phylogenetic relationships of Bacillus species. A phylogenetic tree was constructed by means of neighbor-joining analysis using Bacillus 16S rRNA gene sequences from Korean wild-animal fecal samples (*n* = 18) and NCBI-downloaded strains (*n* = 25). The outgroup strains were Geobacillus stearothermophilus (AY608948.1) and Saccharococcus thermophilus 657 (NR036770.1). The strains isolated in this study have been underlined and are in bold. Bootstrap values lower than 50 were not considered in this phylogenetic tree.

We confirmed that the isolated *Bacillus* species matched the GenBank database as well as phylogenetic analysis (Table 2 and Figure 3). In addition, we successfully isolated, screened, and sequenced all *Bacillus-*positive samples except one (water deer fecal, fecal id: SNHyIn_WD8, colony id: BA#8). Among the 19 colonies, five colonies were from water deer (*H. i. argyropus*) fecal samples, while 13 colonies were from striped field mouse (*A. agrarius*) fecal samples, and one colony from water deer was found to be a chimeric sequence upon sequencing (Table 2).

The 18 PCR products amplified using the 16S rRNA primer sets were sequenced, and the sequences were visually annotated. After sequencing, we recovered the portions of the sequences from approximately 1262 to 1272 bp (Table 2). For identification, these sequences were compared for similarity with the bacterial sequences deposited in GenBank using NCBI BLAST (available on http://www.ncbi.nlm.nih.gov/, accessed on 30 March 2022). The bacterial colonies were identified by means of 99–100% identity, with 99–100% sequence coverage in the sequence-similarity comparison (Table 2).

### 3.3. Development of an SNP-Based Primer for Discrimination between Different Members of the Bacillus cereus Group

We then designed SNP-based 16S rRNA primers to differentiate between the three *Bacillus* species (*B. cereus*, *B. anthracis,* and *B. thuringiensis*). Four SNP positions were detected upon alignment of the four 16S gene sequences of *Bacillus*, which were then used to distinguish between the different *B. cereus* group species. We observed four SNP sites in the aligned gene sequences (‘Y: T > C’; ‘Y: T > C’; ‘M: A > C’; ‘W: A > T’) (Figure S2). An altered base was introduced at the third position of a triplet base at the 3′-end of each primer for efficient discrimination between the three close strains of the *B. cereus* group (Table 1). In the designed SNP-based marker “Bc_F1m/BCR1m”, we included at least two SNPs (one natural and the other an altered base: “R = A or G; Y = C or T”). For instance, the length of the forward primer (BcF1m) was 24 bp, from 522 to 545 bp (the position of the natural SNP was ‘T = 545′, while the transversion-altered SNP site was “A = 543; Y > A”), and the length of the reverse primer (BCR1m) was 21 bp, from 1084 to 1104 bp (the position of the natural SNP was “T = 1084, complementary of A”, and the transversion-altered SNP site was “T = 1086, complementary of A; R > T”). The size of the target-amplified PCR product was approximately 583 bp (Figure S3B). Similarly, another SNP-based primer set was designed (BcF2m/BCR2m), and the size of its target-amplified PCR product was approximately 174 bp (Figure S3C).

### 3.4. Phylogenetic Analysis of Bacillus Species Isolated from Wild-Animal Fecal Samples

In phylogenetic neighbor-joining (NJ) analysis, the 25 *Bacillus* 16S rRNA gene sequences acquired from NCBI and the 18 *Bacillus* 16S rRNA gene sequences acquired from Korean wild-animal fecal samples (this study) clustered well to the three different clades of *Bacillus* species. Among the 18 *Bacillus* strains detected, 12 strains clustered with the *B. amyloliquefaciens* group and five strains clustered with the *B. megaterium* group. The one remaining *Bacillus* strain, BA#01 (MF139612), which was detected in an *A. agrarius* fecal sample, clustered with the *Bacillus cereus* group (Figure 3).

In the phylogenetic tree, we also considered the closely related *Bacillus* strains regarded as outgroups, for example, *Geobacillus stearothermophilus* (AY608948.1) and *Saccharococcus thermophilus* 657 (NR036770.1) (Figure 3). Sequence data analysis, phylogenetic analysis, and genetic sequence variance of *Bacillus* species were conducted using the maximum-likelihood method in MEGA.

**Figure 4:**
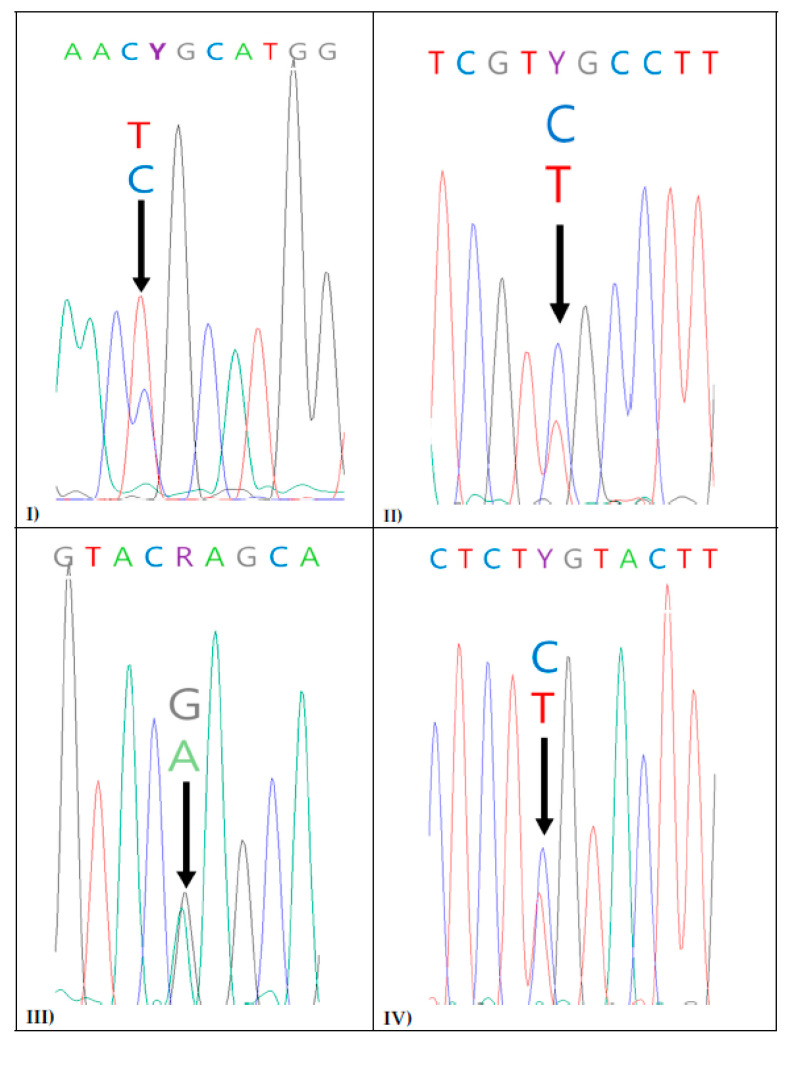
(**I**–**IV**). Representative chromatograms for the 16S rRNA gene sequences of three different *Bacillus* group species. (**I**) *Bacillus cereus* strain BA#1 (Y = T/C exists at position 183 in the reference *B. cereus* strain ATCC 14893 16S rRNA gene, accession no. GQ911551.1). (**II**) *B. amyloliquefaciens* strains BA#12 and BA#19 (Y = C/T exists at position 275 in the reference *B. amyloliquefaciens* strain ATCC 23842 16S rRNA gene, accession no. JF749277). *(***III**) *B. megaterium* strains BA#5 and BA#6 (R = G/A exists at the position 460 in the reference *B. megaterium* strain ATCC 25848 16S rRNA gene, accession no. GQ911553.1). (**IV**) *B. megaterium* strains BA#5 and BA#6 (Y = C/T, exist at the position 473 in the reference *B. megaterium* strain ATCC 25848 16S rRNA gene, accession no. GQ911553.1).

We also observed the pairwise Kimura 2-parameter (K2P) distances for the 18 inter-species relationships among the three different *Bacillus* species (*B. cereus*, *B. amyloliquefaciens*, and *B. megaterium*). The highest genetic variance (0.0701 ± 0004 (*n* = 60, range = 0.0692–0.0701)) was observed within all *Bacillus* species, while the second-highest genetic variance (0.0104 ± 0.0237 (*n* = 77, range = 0–0.659)) was observed between the *B. amyloliquefaciens* and *B. cereus* groups. Pairwise K2P distances (=0) in the boxes indicate that there is no sequence difference between the corresponding inter-species 16S rRNA gene sequences of *Bacillus* in the pairwise comparisons. The numbers in bold indicate interspecies K2P distances (Table S2). Moreover, the sequences were checked carefully, and ambiguous sites—where two different bases were detected at the same position in the sequence—were observed in some *Bacillus* sequences. When the electropherograms were visually observed, the *B. cereus* strain BA#1 had double peaks (T/C, A/G, and C/A) at nucleotide positions 60, 129, and 797, respectively (based on the reference *B. cereus* ATCC 14579 16S rRNA gene; MH281748.1). The representative ambiguous sites are shown in Figure 4.

Moreover, we observed polymorphic sites (*n =* 113), which were detected in the aligned gene sequences of the 18 *Bacillus* strains. A common type of polymorphic mutation observed was a transition mutation R: A > G or G > A (marked using different colors), followed by Y: T > C or C > T (Table S3). In addition, transversion mutations were also observed (A > T, A > C, G > T, and G > C) in the aligned gene sequences.

## 4. Discussion

Wild animals can disseminate zoonotic pathogens that cause different human and animal diseases, especially zoonotic bacteria, including zoonoses caused by *Bacillus* [3,43]. *Bacillus* is a genus of diversified bacteria widely distributed in animal feces [43,44,45] that can cross-contaminate the agricultural environment [44]. In this study, we designed *Bacillus* 16S rRNA-based primers for *Bacillus* species-specific identification. In addition, an SNP-based singleplex marker was also developed in this study for the discrimination of *Bacillus cereus* group-specific bacteria (Table 1).

Chon et al. [46] compared the morphological pattern of *B. cereus* on two specific selective media (MYP and BCSA media) and found the colonies of *B. amyloliquefaciens* bacteria to be yellow in color on MYP agar [47]. In our study, we observed a similar color of *B. cereus* and *B. amyloliquefaciens* strains on MYP agar (Figure S1).

It is difficult to differentiate different strains within a bacterial species due to the microheterogeneity in their 16S rRNA gene sequences, which is common in *Bacillus* as well. Several studies have shown that there is more than 95% sequence identity in the same genus and more than 97% sequence identity in the same species [48,49]. Despite this, 16S rRNA gene sequences and intergenic transcribed sequences have been employed for *Bacillus* identification in several research studies [26,27,50]. In our study, the identified *Bacillus* strains showed approximately 99–100% sequence identity with 99–100% sequence coverage in the sequence-similarity comparison (Table 1).

The use of 16S rRNA molecular markers [51] offers an accurate identification method for bacterial species identification, whereas culture-based identification methods sometimes produce inaccurate results. A previous study showed that the sensitivity and specificity of molecular methods are higher than those of culture-based methods [51]. Another study showed that the prevalence of enteropathogens, specifically in *Salmonella* cases (23 positive out of 24 fecal samples) was higher in conventional tests than that in the molecular tests (21 positive out of 24 fecal samples) [52]. Similarly, in the present study, we identified that 25 animal fecal samples (31.25%) were positive in the culture-based test, while only 19 samples (23.75%) were positive in the molecular tests (Table 2). Nevertheless, bacteria of the *Bacillus cereus* group share the same morphological and biochemical characteristics. Studies involving genome sequences reveal that 16S rRNA gene sequences are highly conserved within the species level [50]. Thus, these highly conserved 16S rRNA gene sequences do not allow for discrimination among the three species of *B. cereus*, *B. anthracis*, and *B. thuringiensis* by means of phylogenetic analysis [53]. In addition, the three species are genetically very close to each other [36]. The sequence similarity of the 16S rRNA gene sequences *of B. anthracis, B. cereus*, and *B. thuringiensis* is very high (>99%) compared to other *B. cereus* group species [36]. They share a large core set of conserved genes and even mix with other *Bacillus* groups in the phylogenetic clusters [30]. Indeed, it has been proposed that *B. anthracis*, *B. cereus*, and *B. thuringiensis* should be considered one species [54].

SNP-based techniques are capable of detecting strains and subspecies [50]. The number of SNPs in the core genome of a reference genome has been used to accurately identify similar species of bacteria [55]. Moreover, a study found that SNPs (polymorphism Y = C/T, which exists at site 173 in the majority of strains of the *B. cereus* group and R = A/G, which exists at site 278 in many strains of the *B. amyloliquefaciens* group) in 16S rRNA gene sequences could help differentiate between similar species of *Bacillus*. It also suggested that specific SNPs could be used to design SNP-based markers [31]. Sacchi et al. [36] previously reported an SNP at a specific position (SNP at position 1137 of 16S rRNA gene sequences) in some *B. cereus* strains [31,56]. Another SNP “R”, at position 1139 of *B. anthracis,* has also been identified [56,57], which could be used for discrimination among close members of the *B. cereus* group [56]. DNA with high-quality sequences and SNP analysis can facilitate bacterial discrimination among very close bacterial species. As per our observation, ambiguous peaks (two peaks) occur in the electropherograms of some *Bacillus* sequences (Figure 4); therefore, visual inspection is necessary for the accurate analysis of 16S rRNA gene sequences [31].

Sacchi et al. (2002) compared members of the *B.* cereus group and found eight SNPs in the 16S rRNA gene sequences from 107 *B. cereus* group species (*B. cereus*, *B. anthracis,* and *B. thuringiensis*) [36]. In our study, we identified four polymorphic nucleotides in the aligned gene sequences of *B. cereus* group strains (*B. cereus*, *B. anthracis*, and *B. thuringiensis*), which were then used to design SNP-based markers for identification of the *B. cereus* group (Figure S2 and Table 1). Thus, we compared *B. cereus*, *B. anthracis,* and *B. thuringiensis* based on the SNP sites in the gene sequences. Fernandez et al. (2006) [31] showed ambiguous codes (R = A/G at position 460 and Y= T/C at position 473) in a reference bacterium, *B. megaterium* ATCC 25848 (accession no. GQ911553). Similarly, we observed the same ambiguous codes (R and Y) at the same position in the laboratory-isolated wildtype strain of *B*. *megaterium* 16S rRNA sequences (BA#5 and BA#6) (Figure 4III). In addition, *B. megaterium* possessed ambiguous dual peaks (A/G at position 460 and T/C at position 473) in two reference bacteria 16S rRNA sequences (*B. megaterium* strains, ATCC-25848 and −14581) [31], and in our study, a similar observation was noted in the *B. megaterium* strains BA#5 and BA#6 (Figure 4III). A previous study showed that dual peaks (A and G) at nucleotide position 194 were unique to *B. amyloliquefeciens* [31], but in the current study, we found this occurrence in only two (dual peaks, A and G at a different position, 275) out of the 10 B. *amyloliquefaciens* strains, based on visual observation of the electropherograms (Figure 4II).

Kuwana et al. [58] showed the discrimination pattern of *B. cereus* group strains using eight primers of random amplified polymorphic DNA (RAPD) markers. In another study, 18 *Bacillus subtilis* strains were identified in domestic animal feces in Korea [45]. Wu et al. identified *Bacillus* isolates from feedlot cattle fecal samples using the RAPD technique [44].

In a study conducted in South Korea, 34 fecal samples from wild mammals were tested for the presence of *B. cereus*. The study revealed that 18 (53%, 18/34) samples were *Bacillus*-positive, but only one sample (8.2%) was detected as *B. cereus* [10]. These results support our present study (Table 2). Moreover, our previous study showed the presence of *Bacillus* in the feces of wild mice [12]. In an indigenous area in Japan, fecal samples from 10 wild Shika deer (*Cervus nippon keramae*) were tested, and 8.5% of them were positive for *B. cereus* [59]. Similarly, in the present study, we found that within the *Bacillus*-positive striped field mouse fecal samples, 6.25% (1/16) were *B. cereus* (Table 2). This sample, which was from a striped field mouse (fecal id: ONApPe_M1) fecal sample, was identified as *B. cereus* strain BA#1 (MF139624).

Several studies have shown that *Bacillus* species (*B. amyloliquefaciens*, *B. velezensis*, *B. subtilis*, *B. siamensis*, and *B. methylotrophicus)* are grouped into a single clade, *B. amyloliquefaciens,* phylogenetically [60,61], which was further proved in this study as a separate clade of *B. amyloliquefaciens*. We concluded that an SNP-based marker could be used for the identification of closely related strains within a *Bacillus* group. In addition, it can also be used to provide inter- and intra-species information. Our study was consistent with previous reports [56] in terms of identifying inter- and intra-species differences among strains belonging to the *Bacillus* species (Table S2). Moreover, the present work describes 113 novel polymorphisms in the conserved region of the 16S rRNA genes in wild-type strains of *Bacillus* detected in wild-animal feces (highlighted using different colors in Table S3). SNP analysis provided more intra-species information within *B. amyloliquefaciens*, *B. cereus,* and *B. megaterium*. Therefore, this work provides valuable information at the inter-species and intra-species levels in a group of *Bacillus* pathogens isolated from wild-animal feces. Moreover, the SNPs identified in this study may help in the design of primers that target the conserved region of the 16S rRNA gene of wild-animal-borne *Bacillus* species, which are widely used for detection and typing purposes. The automated base calling results in the consensus sequence of 16S rRNA genes of closely related members of the *Bacillus cereus* group generated using a basecaller software may lack potentially important information that is available in the electropherograms. SNP analysis of high-quality DNA sequences would facilitate the identification of closely related species.

## 5. Conclusions

*Bacillus* species, specifically *B. cereus,* are genetically heterogeneous and ambiguous in nature. Universal 16S primers fail to differentiate between closely related bacterial species. Therefore, we designed and validated *Bacillus* species-specific 16S rRNA primers and *Bacillus cereus* group-specific SNP-based primers, including *Bacillus cereus, B. anthrax*, and *B. thuringiensis*, for accurate identification. We built a phylogenetic tree and performed genetic analysis to determine the genetic variation within the Korean-isolated wild-type *Bacillus* species. Phylogenetic analysis of *Bacillus* sequences showed the *Bacillus-*specific clusters. SNP knowledge is important for *Bacillus* identification and for designing primers or probes for intra- and inter-specific discrimination of *B. cereus*, *B. megaterium*, and *B. amyloliquefeciens*. However, different pathogenic agents, e.g., *B. cereus*, *B. anthracis,* and other pathogens, could be disseminated from wild-animal feces to agricultural environments, which could be a great threat not only to agricultural workers but also to the general population. They can also cross-contaminate environmental sources such as soil, water, and agricultural leafy vegetables. Ultimately, this results in humans being exposed to these pathogens. Therefore, special attention needs to be paid to develop quick, accurate, and early detection methods to mitigate animal-carried, food-borne pathogen exposure to agricultural areas. This research information can be helpful for initiating the necessary measures against pathogenic *Bacillus* dissemination from wild-animal feces. However, further research should be conducted on samples from diverse sources, such as wild and domestic animals, soil, foods, and so on, on a wide range of *Bacillus* species for evaluation of the efficiency of the *Bacillus* species-specific 16S rRNA markers and *Bacillus cereus*-specific SNP-based markers developed in this study.

## Figures and Tables

**Table 1:** *Bacillus* species-specific ribosomal 16S rRNA primer set and *Bacillus cereus-*specific SNP-based primer set designed using 16S rRNA gene sequences.

Identification	Primer Code	Primer Sequence	Primer Length (bp)	Annealing TEMPERATURE (°C)	PCR Product (bp)	Remarks ^a^
*Bacillus* species-specific ^b^	Ba_F	CGRACGGGTGAGTAACACG	19	58	712	*Bacillus* species-specific markers based on 16S rRNA (sequencing primers)
	Ba_R	GACTACCAGGGTATCTAATCC	21			
	Ba_F1	GGAGGAACACCAGTGGCGAAG	21		678	
	Ba_R1	CCCGGGAACGTATTCACCGC	20			
*Bacillus cereus-*specific	BcF1m	GGGAAGAACAAGTGCTAGTTGYAT	24	62	583	*B. cereus-*specific markers based on SNP-sites (this study); transversion mutation (altered bases)
	BCR1m	GAAGCCCTATCTCTAGGGRTT	21			
	BcF2m	CCAGGTCTTGACATCCTCTYAA	22	65	174	
	BCR2m	GTCACCTTAGAGTGCCCAARTT	22			

^a^ Altered bases (transversion mutated, “R” = A/G; “Y” = C/T) have been marked in red, ^b^ two primer sets (one primer set, Ba_F and Ba_R, and another primer set, Ba_F1 and Ba_R1) were used to identify *Bacillus* species from wild-animal feces (Korean water deer (*Hydropotes inermis argyropus*) and striped field mouse (*Apodemus agrarius*)).

**Table 2:** Culture- and molecular-based identification of *Bacillus* using 16S rRNA gene sequences obtained from wild animal (including water deer (*Hydropotes inermis argyropus*) and striped field mouse (*Apodemus agrarius*)) fecal samples.

Host	Fecal Id	Colony Id	Coverage	Similarity	bp	Accession	Matched Bacteria from NCBI (Accession No)	Bacillus Group
*Apodemus agrarius*	ONApPe_M1	BA#01	99	99	1262	MF139612	*Bacillus cereus* ZLynn800-11 (KY316431.1)	*B. cereus*
	ONApPe_M2	BA#02	100	100	1262	MF139613	*B. siamensis* FL11 (KY818929.1)	*B. amyloliquefaciens*
	ONApPe_M3	BA#03	100	100	1267	MF139615	*B. siamensis* KCTC 13613 (AJVF01000043)	
	ONApPe_M10	BA#10	100	99	1268	MF139620	*B. amyloliquefaciens* HY-5 (KY886133.1)	
	ONApPe_M11	BA#11	100	99	1267	MF139619	*B. amyloliquefaciens* IIHR (OL477453.1)	
	ONApPe_M13	BA#13	100	100	1268	MF139622	*B. amyloliquefaciens* HY-5 (KY886133.1)	
	ONApPe_M14	BA#14	99	100	1268	MF139616	*B. amyloliquefaciens* H5Y (KY886133.1)	
	ONApPe_M15	BA#15	100	99	1267	MF139617	*B. amyloliquefaciens* MN4 (KY305419.1)	
	ONApPe_M18	BA#18	100	100	1268	MF139614	*B. velezensis* GQJK49 (KY952710.1)	
	ONApPe_M19	BA#19	100	100	1267	MF139618	*B. amyloliquefaciens* 2M5-2 (KX267874.1)	
	ONApPe_M33	BA#33	100	99	1266	MF139621	*B. amyloliquefaciens* IIHR (OL477453.1)	
	ONApPe_M38	BA#38	100	99	1272	MF139623	*B. amyloliquefaciens* MN4 (KY305419.1)	
	ONApPe_M39	BA#39	100	100	1267	MF139624	*B. amyloliquefaciens* HY-5 (KY886133.1)	
	ONApPe_M18	BA#18	-	-	-	-	-	Not found
	ONApPe_M20	BA#20	-	-	-	-	-	
	ONApPe_M37	BA#37	-	-	-	-	-	
*Hydropotes inermis argyropus*	SNHyIn_WD4	BA#04	100	100	1267	MF139625	*Bacillus sp.* JDMASP69 (KX817970.1)	*B. megaterium*
	SNHyIn_WD5	BA#05	100	100	1270	MF139627	*B. megaterium* JDMARP68 (KX817907.1)	
	SNHyIn_WD6	BA#06	100	100	1268	MF139629	*B. megaterium* T11-11.1 (OM062585.1)	
	SNHyIn_WD7	BA#07	100	99	1270	MF139628	*B. megaterium* strain ZLynn800-19 (KY316435.1)	
	SNHyIn_WD8	BA#08	x	x	x	x	Chimeric	
	SNHyIn_WD9	BA#09	100	100	1269	MF139626	*B. megaterium* 1000-18 (KY316449.1)	
	SNHyIn_WD10	BA#10	-	-	-	-	-	Not found
	SNHyIn_WD16	BA#16	-	-	-	-	-	
	SNHyIn_WD25	BA#25	-	-	-	-	-	

“x” = could be amplified with the 16S rRNA primers, but the sequence was chimeric; therefore, there was no match in the NCBI database; “-” = no amplification with the 16S rRNA primers, but positive in the culture.

## Data Availability

The genetic sequence (16S rRNA) data from this study were deposited to the NCBI with accession no. MF139612–MF139629.

## Supplementary Materials

The following are available online at https://www.mdpi.com/article/10.3390/ani12080979/s1, Figure S1: A. The colony of *Bacillus cereus,* BA_1(MF139612), on *Bacillus cereus* Selective Agar (BCSA) was observed to be a distinctive turquoise to peacock blue color. B. Microscopic view showed a distinctive egg yolk-like precipitate, which has been marked using an arrow. C. The colonies of *Bacillus cereus,* BA#1 (MF139612), on Mannitol Egg Yolk Polymixin Agar (MYP) were of pink or pink-orange color, with white precipitation around the *Bacillus* colonies. D. Microscopic view of positive *Bacillus cereus* with whitish precipitation. E. Yellow colonies of *B. amyloliquefaciens*, BA#17 (MF139624), observed on MYP agar. F. Microscopic view of *B. amyloliquefaciens* group of bacteria. G. The white amorphous, large colonies of *Bacillus* cereus grown on Nutrient Agar plate (NA). H. Control media of MYP agar plate without inoculation of bacterial culture from fecal sample. Figure S2: Single nucleotide polymorphism (SNP)-based primer was developed based on natural SNPs and modified nucleotide bases on the aligned 16S gene sequences of the *Bacillus cereus* group (*B. cereus*, *B. anthracis*, and *B. thuringiensis*). Natural SNPs were marked by red color with red square shape, target mutated bases marked by black square shape, and primer pairs were indicated by underline shaded. In this figure, *Bacillus cereus* strain B.1 (MF139612) was detected from a striped field mouse (*Apodemus agrarius*) fecal, and the other three species were downloaded from NCBI GenBank. Figure S3: PCR amplification of 16S primer (16S rRNA, Ba_F/Ba_R1) was used for amplification of 19 *Bacillus* species from wild animal fecal [upon using the first (Ba_F/R) sequencing primer of forward primer, Ba_F and second (Ba_F1/R1) sequencing primer of reverse primer, Ba_R1), see in the Table 1]. B. SNP-based primer (BcF1m and BCR1m) was developed by natural and artificial mutated bases at the 3‘ end of triplet bases of each primer C. Similarly, SNP-based (BcF2m/BCR2m) primer was developed for *Bacillus cereus* group specific identification. ‘M’ indicate DNA 100bp marker, The gel lane numbers are as follows: (accession no. of isolated *Bacillus* strains, 1–19) No.1 = MF139612; No.2 = MF139613; No.3 = MF139615; No.4 = MF139625; No.5 = MF139627; No.6 = MF139629; No.7 = MF139628; No.8 = chimeric sequence (No Accession No); No.9 = MF139626; No.10 = MF139620; No.11 = MF139619; No.12 = MF139621; No.13 = MF139622; No.14 = MF139616; No.15 = MF139617; No.16= MF139623; No.17 = MF139624; No.18 = MF139614; No.19 = MF139618; and non-*Bacillus* sp. lane no I-V) No.I = *Salmonella enterica* (NCCP-15756); No.II = *Escherichia coli* (NCCP-14034); No.III = *Shigella dysenteriae* (NCCP-14746), No.IV = *Enterobacter cloacae* (10173), No.V = *Pseudomonas aeruginosa* (NCCP-16099) and No.VI = only PCR mixture without sample DNA as negative control. Table S1: List of fecal samples with the collection locality, animal species, and Global Positioning System (GPS) information; Table S2: Pairwise Kimura 2-Parameter (K2P) distances 18 interspecific relationships of three different Bacillus group. Table S3: Polymorphic sites (SNPs, n = 113) of 18 Bacillus 16S gene sequences of three different Bacillus group.
