# Supplementary material for: Molecular Identification of Bacillus Isolated from Korean Water Deer (Hydropotes inermis argyropus) and Striped Field Mouse (Apodemus agrarius) Feces by Using an SNP-Based 16S Ribosomal Marker
Source: Animals (Basel). 2022 Apr 10;12(8):979. doi: 10.3390/ani12080979 (PMC9031142; doi:10.3390/ani12080979)
Supplement: Supplementary file 1 [file animals-12-00979-s001.zip › Figure S1..pptx]

## Slide 1
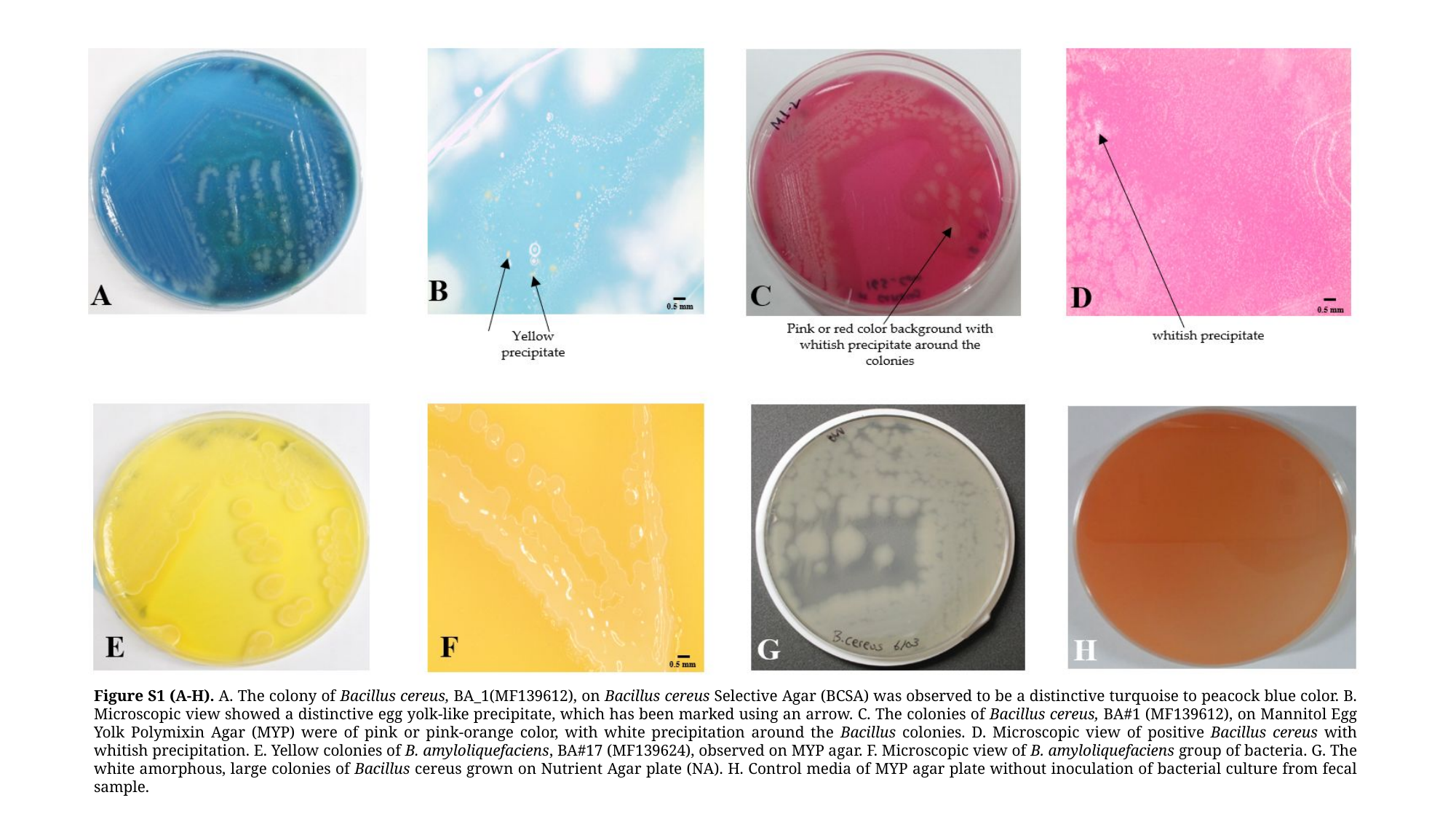

Figure S1 (A-H). A. The colony of Bacillus cereus, BA_1(MF139612), on Bacillus cereus Selective Agar (BCSA) was observed to be a distinctive turquoise to peacock blue color. B. Microscopic view showed a distinctive egg yolk-like precipitate, which has been marked using an arrow. C. The colonies of Bacillus cereus, BA#1 (MF139612), on Mannitol Egg Yolk Polymixin Agar (MYP) were of pink or pink-orange color, with white precipitation around the Bacillus colonies. D. Microscopic view of positive Bacillus cereus with whitish precipitation. E. Yellow colonies of B. amyloliquefaciens, BA#17 (MF139624), observed on MYP agar. F. Microscopic view of B. amyloliquefaciens group of bacteria. G. The white amorphous, large colonies of Bacillus cereus grown on Nutrient Agar plate (NA). H. Control media of MYP agar plate without inoculation of bacterial culture from fecal sample.
