# Supplementary material for: Molecular Identification of Bacillus Isolated from Korean Water Deer (Hydropotes inermis argyropus) and Striped Field Mouse (Apodemus agrarius) Feces by Using an SNP-Based 16S Ribosomal Marker
Source: Animals (Basel). 2022 Apr 10;12(8):979. doi: 10.3390/ani12080979 (PMC9031142; doi:10.3390/ani12080979)
Supplement: Supplementary file 1 [file animals-12-00979-s001.zip › Figure S2..pptx]

## Slide 1
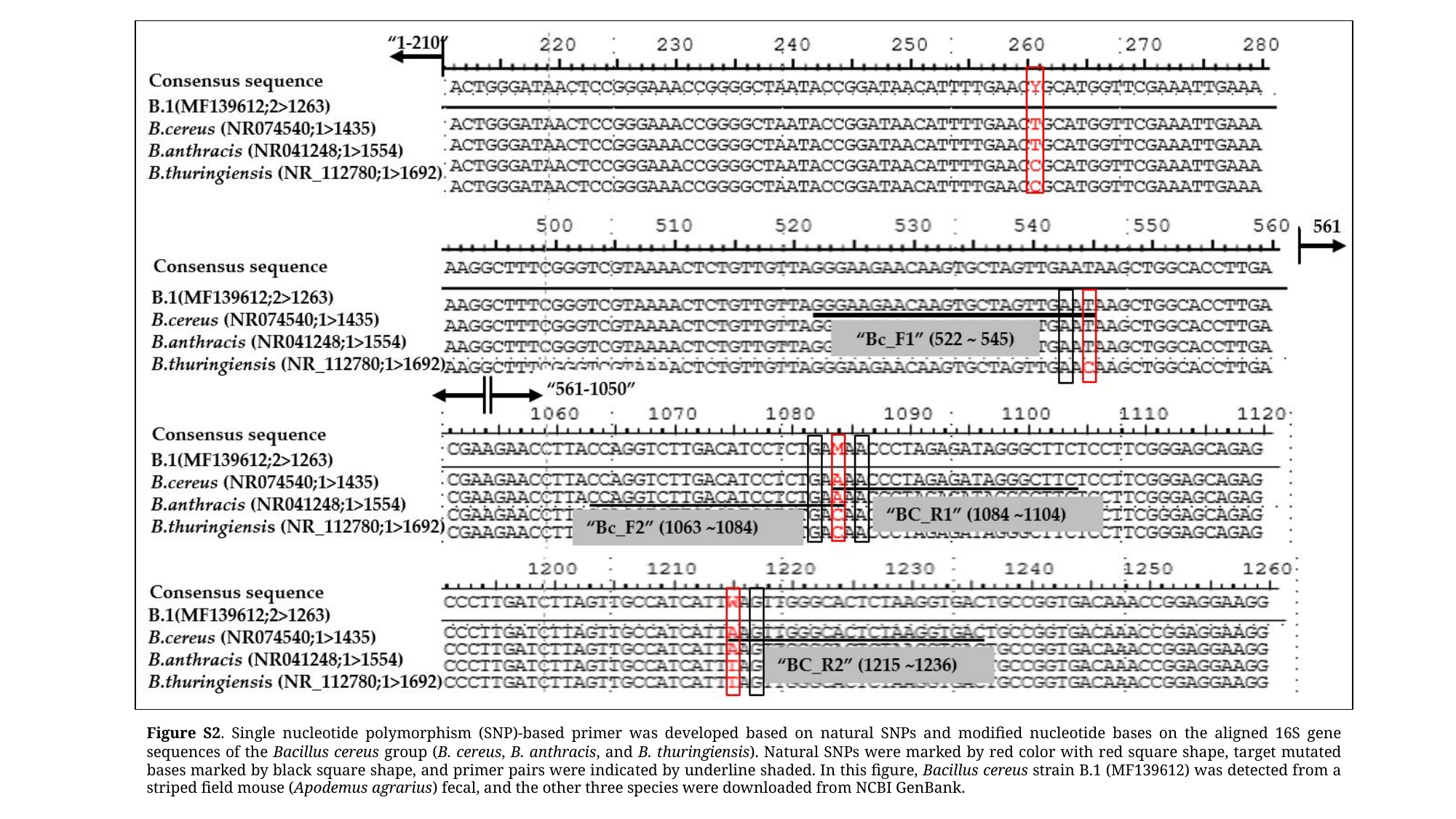

Figure S2. Single nucleotide polymorphism (SNP)-based primer was developed based on natural SNPs and modified nucleotide bases on the aligned 16S gene sequences of the Bacillus cereus group (B. cereus, B. anthracis, and B. thuringiensis). Natural SNPs were marked by red color with red square shape, target mutated bases marked by black square shape, and primer pairs were indicated by underline shaded. In this figure, Bacillus cereus strain B.1 (MF139612) was detected from a striped field mouse (Apodemus agrarius) fecal, and the other three species were downloaded from NCBI GenBank.
