# Supplementary material for: Molecular Identification of Bacillus Isolated from Korean Water Deer (Hydropotes inermis argyropus) and Striped Field Mouse (Apodemus agrarius) Feces by Using an SNP-Based 16S Ribosomal Marker
Source: Animals (Basel). 2022 Apr 10;12(8):979. doi: 10.3390/ani12080979 (PMC9031142; doi:10.3390/ani12080979)
Supplement: Supplementary file 1 [file animals-12-00979-s001.zip › Figure S3..pptx]

## Slide 1
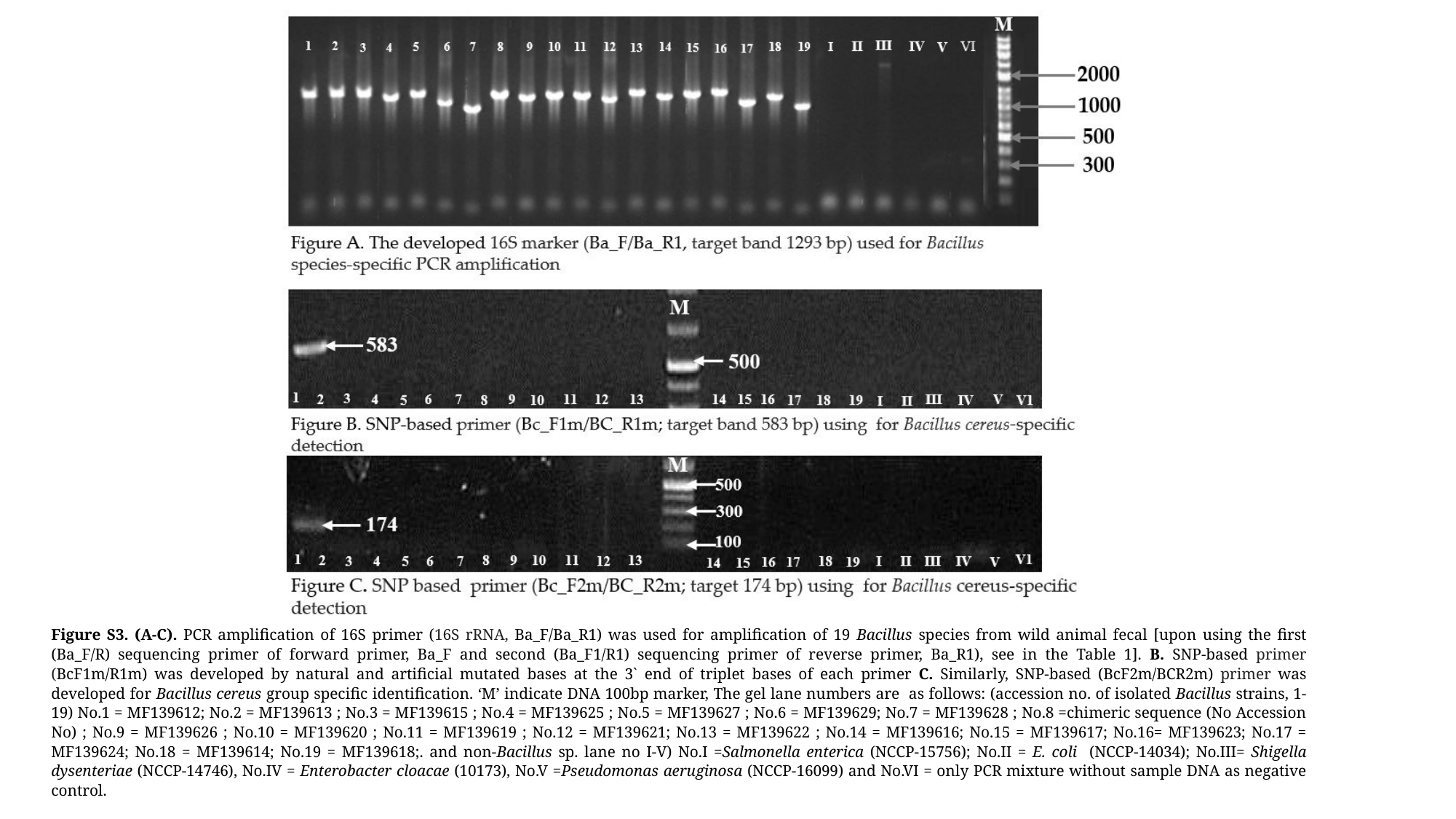

Figure S3. (A-C). PCR amplification of 16S primer (16S rRNA, Ba_F/Ba_R1) was used for amplification of 19 Bacillus species from wild animal fecal [upon using the first (Ba_F/R) sequencing primer of forward primer, Ba_F and second (Ba_F1/R1) sequencing primer of reverse primer, Ba_R1), see in the Table 1]. B. SNP-based primer (BcF1m/R1m) was developed by natural and artificial mutated bases at the 3` end of triplet bases of each primer C. Similarly, SNP-based (BcF2m/BCR2m) primer was developed for Bacillus cereus group specific identification. ‘M’ indicate DNA 100bp marker, The gel lane numbers are as follows: (accession no. of isolated Bacillus strains, 1-19) No.1 = MF139612; No.2 = MF139613 ; No.3 = MF139615 ; No.4 = MF139625 ; No.5 = MF139627 ; No.6 = MF139629; No.7 = MF139628 ; No.8 =chimeric sequence (No Accession No) ; No.9 = MF139626 ; No.10 = MF139620 ; No.11 = MF139619 ; No.12 = MF139621; No.13 = MF139622 ; No.14 = MF139616; No.15 = MF139617; No.16= MF139623; No.17 = MF139624; No.18 = MF139614; No.19 = MF139618;. and non-Bacillus sp. lane no I-V) No.I =Salmonella enterica (NCCP-15756); No.II = E. coli (NCCP-14034); No.III= Shigella dysenteriae (NCCP-14746), No.IV = Enterobacter cloacae (10173), No.V =Pseudomonas aeruginosa (NCCP-16099) and No.VI = only PCR mixture without sample DNA as negative control.
