# Supplementary material for: Molecular Identification of Bacillus Isolated from Korean Water Deer (Hydropotes inermis argyropus) and Striped Field Mouse (Apodemus agrarius) Feces by Using an SNP-Based 16S Ribosomal Marker
Source: Animals (Basel). 2022 Apr 10;12(8):979. doi: 10.3390/ani12080979 (PMC9031142; doi:10.3390/ani12080979)
Supplement: Supplementary file 1 [file animals-12-00979-s001.zip › Supplementary data_legend.pdf]

Supplementary Figure legends

Figure S1 (A-H). A. The colony of *Bacillus cereus,* BA_1(MF139612), on *Bacillus cereus* Selective Agar (BCSA) was observed to be a distinctive turquoise to peacock blue color. B. Microscopic view showed a distinctive egg yolk-like precipitate, which has been marked using an arrow. C. The colonies of *Bacillus cereus,* BA#1 (MF139612), on Mannitol Egg Yolk Polymixin Agar (MYP) were of pink or pink-orange color, with white precipitation around the *Bacillus* colonies. D. Microscopic view of positive *Bacillus cereus* with whitish precipitation. E. Yellow colonies of *B. amyloliquefaciens*, BA#17 (MF139624), observed on MYP agar. F. Microscopic view of *B. amyloliquefaciens* group of bacteria. G. The white amorphous, large colonies of *Bacillus* cereus grown on Nutrient Agar plate (NA). H. Control media of MYP agar plate without inoculation of bacterial culture from fecal sample.

Figure S2. Single nucleotide polymorphism (SNP)-based primer was developed based on natural SNPs and modified nucleotide bases on the aligned 16S gene sequences of the *Bacillus cereus* group (*B. cereus, B. anthracis*, and *B. thuringiensis*). Natural SNPs were marked by red color with red square shape, target mutated bases marked by black square shape, and primer pairs were indicated by underline shaded. In this figure, *Bacillus cereus* strain B.1 (MF139612) was detected from a striped field mouse (*Apodemus agrarius*) fecal, and the other three species were downloaded from NCBI GenBank.

Figure S3. (A-C). PCR amplification of 16S primer (16S rRNA, Ba_F/Ba_R1) was used for amplification of 19 *Bacillus* species from wild animal fecal [upon using the first (Ba_F/R) sequencing primer of forward primer, Ba_F and second (Ba_F1/R1) sequencing primer of reverse primer, Ba_R1), see in the Table 1]. **B.** SNP-based primer (BcF1m and BCR1m) was developed by natural and artificial mutated bases at the 3` end of triplet bases of each primer **C.** Similarly, SNP-based (BcF2m/BCR2m) primer was developed for *Bacillus cereus* group specific identification. 'M' indicate DNA 100bp marker, The gel lane numbers are as follows: (accession no. of isolated *Bacillus* strains, 1-19) No.1 = MF139612; No.2 = MF139613 ; No.3 = MF139615 ; No.4 = MF139625 ; No.5 = MF139627 ; No.6 = MF139629; No.7 = MF139628 ; No.8 =chimeric sequence (No Accession No) ; No.9 = MF139626 ; No.10 = MF139620 ; No.11 = MF139619 ; No.12 = MF139621; No.13 = MF139622 ; No.14 = MF139616; No.15 = MF139617; No.16= MF139623; No.17 = MF139624; No.18 = MF139614; No.19 = MF139618;. and non-*Bacillus* sp. lane no I-V) No.I =*Salmonella enterica* (NCCP-15756); No.II = *Escherichia coli* (NCCP-14034); No.III= *Shigella dysenteriae* (NCCP-14746), No.IV = *Enterobacter cloacae* (10173), No.V =*Pseudomonas aeruginosa* (NCCP-16099) and No.VI = only PCR mixture without sample DNA as negative control.

Supplementary Table legend

Table S1. List of fecal samples with the collection locality, animal species, and Global Positioning System (GPS) information

Table S2. Pairwise Kimura 2-Parameter (K2P) distances 18 interspecific relationships of three different *Bacillus* group.

Table S3. Polymorphic sites (SNPs, n=113) of 18 *Bacillus* 16S gene sequences of three different *Bacillus* group
